# Preventable drivers of acute respiratory infections among under-five children in India’s high-risk zones: application of population attributable fractions analyses

**DOI:** 10.1186/s41182-026-01107-1

**Published:** 2026-11-09

**Authors:** U. Venkatesh, Margubur Rahaman, R. Durga, Moslem Hossain, Om Prakash Bera, Ashoo Grover

**Affiliations:** 1 Department of Community Medicine & Family Medicine, All India Institute of Medical Sciences Gorakhpur, Gorakhpur, Uttar Pradesh 273008 India; 2 Independent Researcher, Gorakhpur, Uttar Pradesh 273008 India; 3 Global Health Advocacy Incubator (GHAI), Washington, DC 20005 USA; 4 Indian Council of Medical Research, Ansari Nagar, New Delhi, 110029 India

**Keywords:** Acute respiratory infection, Under-five children, Spatial epidemiology, Population attributable fraction, Diarrhoeal disease, India, NFHS-5

## Abstract

**Background:**

Acute respiratory infections (ARI) remain a major contributor to morbidity and mortality among under-five children in India. Although previous national studies have identified individual-level risk factors, evidence on the spatial distribution of ARI and the burden statistically attributable to selected exposures within hotspot regions remains limited.

**Methods:**

We analysed data from the 2019–21 National Family Health Survey (NFHS-5) Children’s Recode file using a two-stage analytical framework. First, district-level ARI hotspots were identified among 221,849 under-five children across 707 districts. Second, analyses were restricted to children residing in hotspot districts (weighted *n* = 108,470). Survey-weighted prevalence, multivariable logistic regression, and population attributable fraction (PAF) analyses accounted for sampling weights, clustering, and stratification.

**Findings:**

A total of 167 districts were identified as ARI hotspots, predominantly in central and eastern India. The unrestricted national weighted prevalence of ARI in the study dataset was 2.74%, compared with 7.23% (95% CI 7.08–7.39) within hotspot districts. Recent diarrhoeal illness (AOR 4.37, 95% CI 4.01–4.76) and maternal tobacco use (AOR 1.29, 95% CI 1.06–1.57) were independently associated with ARI. The PAF was 19.6% for recent diarrhoea and 0.7% for maternal tobacco use. Rural residence, interpreted as a contextual marker rather than a directly modifiable exposure, had a model-based PAF of 10.2%. The joint counterfactual scenario yielded a PAF of 28.7% (95% CI 20.4–36.1).

**Conclusions:**

Under-five ARI is geographically concentrated in distinct hotspot districts. Integrated interventions addressing diarrhoeal disease and maternal tobacco exposure, together with improvements in rural child-health services and infrastructure, may reduce ARI burden in these districts. Because rural residence is not itself an intervention, its PAF should be interpreted as a priority-setting indicator of place-based disadvantage rather than as the effect of relocating rural populations.

## Introduction

Acute respiratory infections (ARIs) remain a leading cause of morbidity and mortality among children younger than 5 years worldwide. ARIs account for approximately 15% of all under-five deaths globally, translating to more than 700,000 largely preventable deaths each year [1]. Lower respiratory infections alone contribute to more than 340 million episodes and 2.2 million deaths annually, with over 70% of this burden concentrated in low- and middle-income countries [2]. South Asia and sub-Saharan Africa together account for more than two-fifths of under-five mortality attributable to ARIs. India bears a particularly high burden, underscoring the need for geographically targeted prevention.

Despite this substantial burden, evidence on the geographic distribution and drivers of childhood ARIs in India remains limited [3] Existing studies have predominantly relied on non-spatial analytical approaches, focusing on individual-level risk factors, such as child undernutrition, recent morbidity, household environmental exposures, and maternal tobacco use [4–6]. While these studies have identified important correlates, they have not accounted for spatial dependence in ARI risk or examined whether these associations differ in areas, where ARI burden is concentrated. Consequently, the absence of hotspot-specific evidence has constrained the development of geographically targeted interventions and limited the effectiveness of national child-health programmes.

Moreover, no previous study in India has quantified PAFs for selected ARI-associated exposures within empirically identified hotspot regions [4, 6–10]. PAFs can help estimate the proportion of disease burden that might be avoided under explicitly defined counterfactual scenarios, but causal interpretation requires strong assumptions, including correct model specification, consistency, and absence of residual confounding. This distinction is especially important for contextual characteristics, such as rural residence, which are not directly modifiable interventions.

In this context, the present study applies a multistage spatial epidemiological framework using nationally representative NFHS-5 data. First, we identify district-level hotspots of under-five ARI prevalence across India. Second, the present study examines socio-demographic, maternal, nutritional, and household determinants of ARIs within hotspot regions using survey-weighted regression models. Third, we estimate the PAFs of key modifiable exposures to quantify their potential population-level impact. By integrating spatial analytics with conventional epidemiological methods, this study provides novel, hotspot-specific evidence to inform targeted interventions. The findings have direct relevance for India’s child health and nutrition programmes and contribute to global efforts to achieve Sustainable Development Goal 3.2, which aims to end preventable deaths of children younger than 5 years by 2030.

## Methods

### Data

This study used data from the National Family Health Survey (NFHS-5), conducted in India during 2019–21 [11]. NFHS-5 is a nationally representative, cross-sectional household survey implemented under the Demographic and Health Survey programme, employing a stratified, multistage cluster sampling design. We analysed data from the children’s recode (KR) file, which includes detailed information on health, nutrition, and care practices for children younger than 5 years [11].

**Fig. 1:**
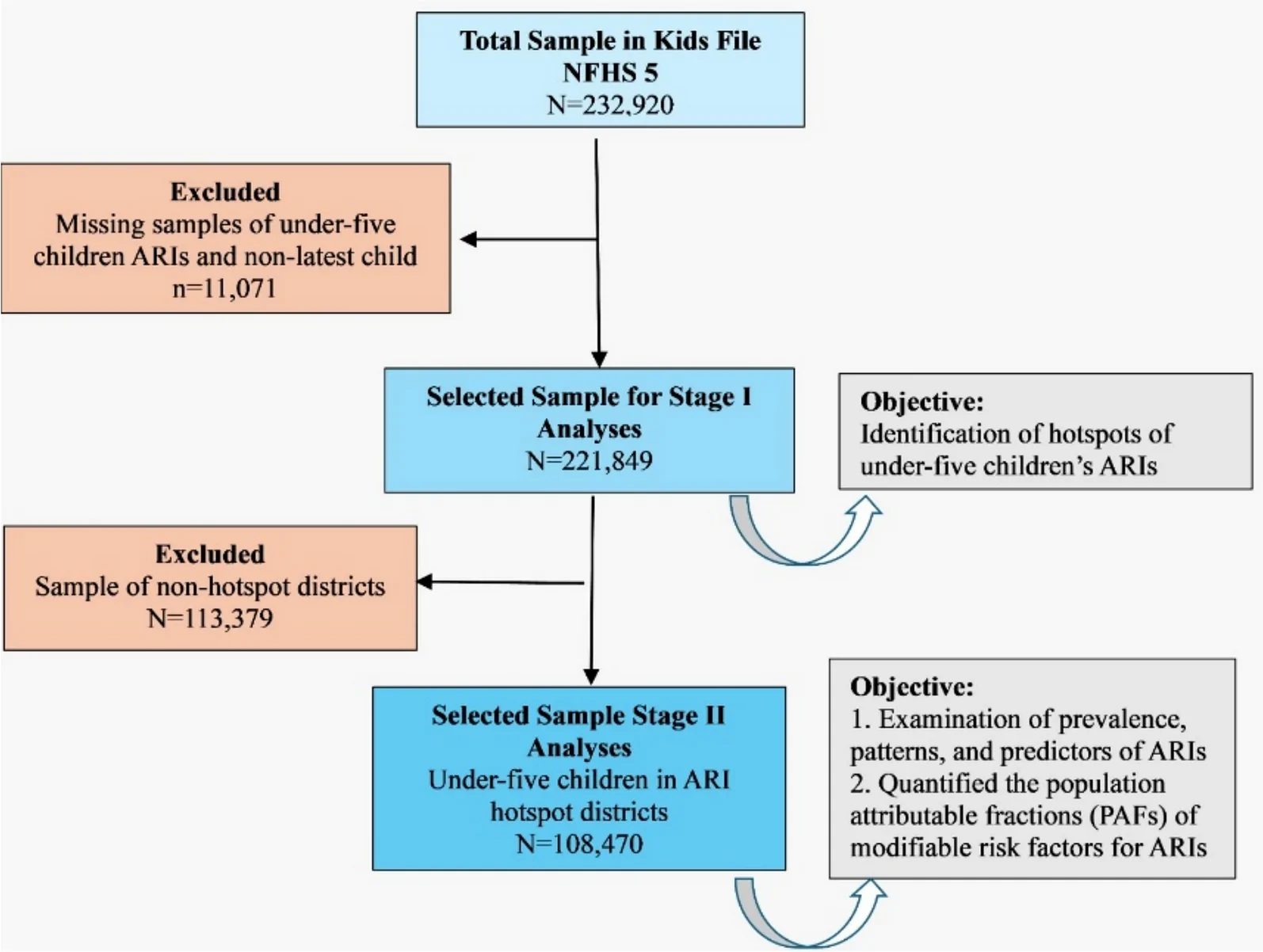
Study sample selection from the National Family Health Survey 2019–21

The analysis was undertaken in two stages. First, 221,849 under-five children with complete ARI information were included in district-level hotspot identification across 707 districts. The survey-weighted national ARI prevalence in this unrestricted sample was 2.74%, consistent with the 2.8% reported in an independent NFHS-5 analysis using the same symptom definition [8]. Second, the sample was restricted to children residing in identified hotspot districts. This yielded a weighted sample of 108,470 children for descriptive analysis. Regression models used complete cases for the covariates entered at each stage: Model I included a weighted *n* of 108,470, whereas Models II and III included a weighted *n* of 96,192 after excluding children with missing anthropometric measurements. Figure 1 summarises sample selection.

### Study variables

The outcome was ARI among under-five children, defined using the standard NFHS symptom-based measure: maternal report of cough accompanied by short, rapid breathing or difficult breathing that was chest-related during 2 weeks preceding the survey. The outcome was coded from the relevant NFHS-5 child morbidity items and applied identically in the national and hotspot-restricted analyses [4, 6, 8, 12, 13].

Explanatory variables were selected a priori based on existing evidence and conceptual frameworks linking child health, maternal factors, and household environment to respiratory morbidity [6, 7, 14]. Child-level characteristics included age (< 12 months, 12–23 months, 24–35 months, 36–47 months, and 48–59 months), sex (male, female), recent diarrhoea (no, yes), and nutritional status (stunting, wasting, and underweight), derived from anthropometric Z-scores using WHO growth standards. Maternal characteristics included tobacco use (smoked or smokeless) and highest educational attainment (no education, primary, secondary, and higher). Household-level variables comprised stool disposal practice (safe or unsafe), type of cooking fuel (clean or unclean), household wealth quintiles (1st, 2nd, 3rd, 4th, and 5th quintile), and place of residence (urban or rural). All variables were categorised following NFHS guidelines and consistent with prior studies examining socioeconomic and environmental determinants of childhood ARI in low- and middle-income settings [7, 11, 15]

### Statistical analysis

We used the Getis-Ord Gi* statistic in ArcGIS 10.8.2 to identify statistically significant spatial concentrations of high and low district-level ARI prevalence. District prevalence estimates were analysed with a spatial-weighted matrix, and standardised Gi* z scores and associated *p* values were used to classify statistically significant hotspots and cold spots:

$${G}_{i}^{*}=\frac{{\sum}_{j=1}^{n}{w}_{i,j}{x}_{j}\stackrel{-}{-X}{\sum}_{j=1}^{n}{w}_{i,j}}{s\sqrt{\frac{n{\Sigma}_{j=1 {w}_{i,j}^{2}}^{n}-({\Sigma}_{j=1 {w}_{i,j}}^{n})2}{n-1}}}$$

where *x*_*j*_ represents the attribute value at location *j*, *w*_*i,j*_ is the spatial weight between locations *i* and *j*, and Xˉ is the mean of the attribute. Standardised z scores were used to classify significant clusters.

We accounted for the complex NFHS design using sampling weights, primary sampling units, and stratification. Descriptive results are presented as weighted percentages with 95% confidence intervals (CIs), estimated using logit-transformed survey-adjusted proportions. Differences in ARI prevalence were assessed using Rao–Scott survey-adjusted chi-square tests. The unrestricted national prevalence was estimated with the same outcome coding and survey specification used for the hotspot-restricted analysis.

Survey-weighted logistic regression estimated associations between ARI and child, maternal, and household characteristics, reported as adjusted odds ratios (AORs) with 95% CIs. Model I included child characteristics; Model II added maternal and nutritional factors; and Model III added household socioeconomic and environmental factors. Complete-case analysis was used for each model. Consequently, the weighted analytic samples were 108,470 for Model I and 96,192 for Models II and III. Multicollinearity was evaluated using variance inflation factors (VIFs).

Adjusted PAFs and 95% CIs were estimated after the fully adjusted survey-weighted logistic model by counterfactual marginal standardisation[16]. For each scenario, predicted ARI probabilities were averaged under the observed exposure distribution and after setting the specified exposure or exposures to the reference level for every child while retaining all other covariates at their observed values. The PAF was calculated as (Pobserved–Pcounterfactual)/Pobserved. For the joint scenario, recent diarrhoea, maternal tobacco use, and rural residence were set simultaneously to their reference levels in a single prediction step; the component PAFs were not added. Confidence intervals incorporated model-estimation uncertainty. Rural residence was retained as a contextual priority-setting indicator, not as a directly modifiable exposure. All analyses were performed in Stata 17.1; maps and figures were produced using ArcGIS 10.8.2, GeoDa 1.20, and R 4.3.1.

## Results

### Hotspots of under-five children’s ARI

**Fig. 2:**
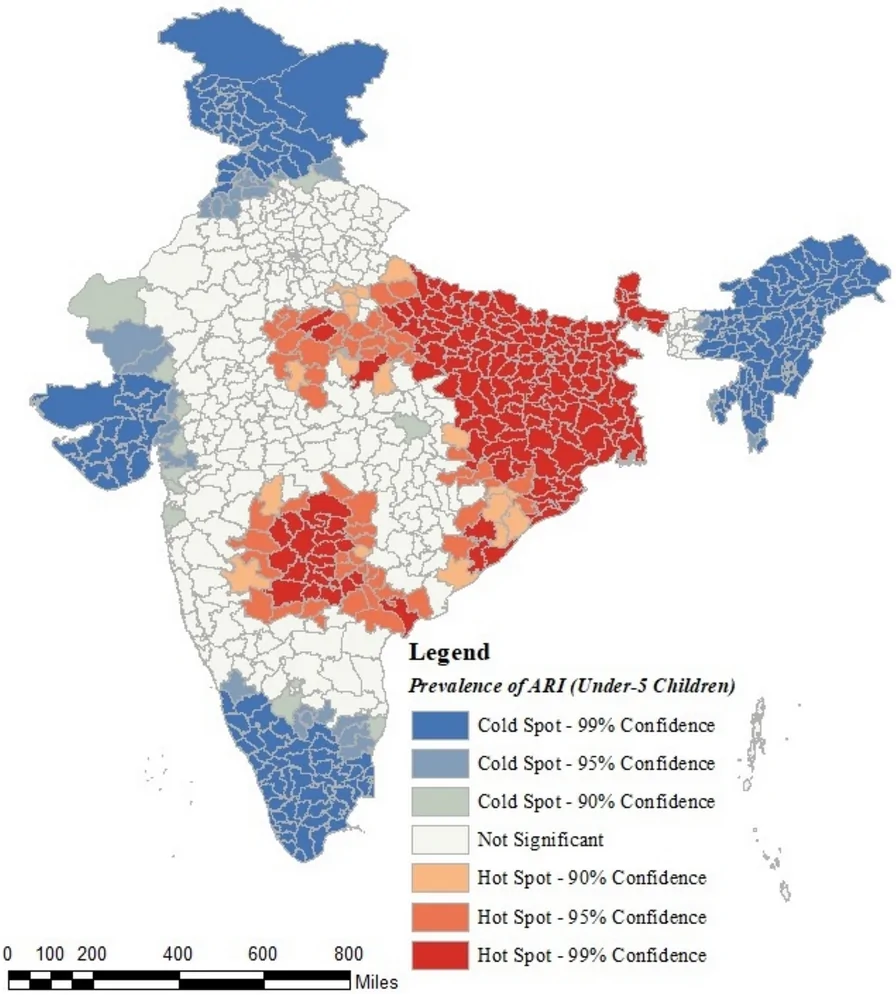
Getis-Ord Gi* map of under-five ARI prevalence across 707 districts, India, 2019–21

A total of 167 districts were identified as statistically significant ARI hotspots (Fig. 2). They were concentrated mainly across central and eastern India, including districts in Bihar, Jharkhand, Odisha, Chhattisgarh, West Bengal, Madhya Pradesh, and parts of Telangana, Andhra Pradesh, and Karnataka.

### Background characteristics of the study population

The survey-weighted national prevalence of ARI was 2.74% in the unrestricted NFHS-5 sample and increased to 7.23% (95% CI 7.08–7.39) among the 108,470 weighted children residing in hotspot districts (Table 1). The approximately 2.6-fold difference is expected, because the second-stage sample was deliberately restricted to districts with statistically elevated ARI prevalence; it does not represent a competing national estimate. The national estimate closely agrees with the 2.8% reported by Varghese and Muhammad using NFHS-5[8].

**Table 1:** Background characteristics of the study population in ARI hotspot regions, India, 2019–21

Characteristics	Weighted N	%	95% CI
Acute respiratory infection (ARI)			
No	100,625	92 · 77	92 · 61–92 · 92
Yes	7,845	7 · 23	7 · 08–7 · 39
Age of child (months)			
< 12	21,810	20 · 11	19 · 87–20 · 35
12–23	21,116	19 · 47	19 · 23–19 · 70
24–35	21,576	19 · 89	19 · 65–20 · 13
36–47	21,378	19 · 71	19 · 47–19 · 95
48–59	22,591	20 · 83	20 · 59–21 · 07
Sex of child			
Male	56,514	52 · 10	51 · 80–52 · 40
Female	51,956	47 · 90	47 · 60–48 · 20
Recent diarrhoea			
No	98,991	91 · 26	90 · 92–91 · 43
Yes	9,479	8 · 74	8 · 57–9 · 08
Stool disposal practice			
Safe	18,743	17 · 28	17 · 06–17 · 51
Unsafe	89,727	82 · 72	82 · 49–82 · 94
Stunting			
Not stunted	59,891	55 · 21	54 · 92–55 · 51
Stunted	38,219	35 · 23	34 · 95–35 · 52
Missing	10,360	9 · 55	9 · 38–9 · 73
Wasting			
Not wasted	76,313	70 · 35	70 · 08–70 · 63
Wasted	19,879	18 · 33	18 · 10–18 · 56
Missing	12,278	11 · 32	11 · 13–11 · 51
Underweight			
Not underweight	64,764	59 · 71	59 · 41–60 · 00
Underweight	35,454	32 · 69	32 · 41–32 · 97
Missing	8,252	7 · 61	7 · 45–7 · 77
Maternal tobacco use			
No	105,630	97 · 38	97 · 29–97 · 48
Yes	2,840	2 · 62	2 · 52–2 · 71
Maternal education			
No education	30,110	27 · 76	27 · 49–28 · 03
Primary	13,178	12 · 15	11 · 96–12 · 34
Secondary	49,711	45 · 83	45 · 53–46 · 13
Higher	15,472	14 · 26	14 · 06–14 · 47
Household wealth quintile			
1st quintile	33,206	30 · 61	30 · 34–30 · 89
2nd quintile	25,445	23 · 46	23 · 21–23 · 71
3rd quintile	19,349	17 · 84	17 · 61–18 · 07
4th quintile	16,982	15 · 66	15 · 44–15 · 87
5th quintile	13,489	12 · 44	12 · 24–12 · 63
Type of cooking fuel			
Clean	47,850	44 · 11	43 · 82–44 · 41
Unclean	60,621	55 · 89	55 · 59–56 · 18
Place of residence			
Urban	24,588	22 · 67	22 · 42–22 · 92
Rural	83,882	77 · 33	77 · 08–77 · 58
Total	108,470	100 · 00	–

Values are weighted using sampling weights. Percentages are presented with 95% confidence intervals estimated using logit-transformed survey-weighted proportions

### Prevalence of ARI by background characteristics

In ARI hotspot regions of India, the weighted prevalence of ARI among under-five children was 7.2% (95% CI 7.08–7.38) during 2019–2021 (Table 2). The greatest differences in ARI prevalence were observed by recent diarrhoeal illness, age, and place of residence. Children who experienced recent diarrhoea had a substantially higher prevalence of ARI than those without recent diarrhoea (22.5% vs 5.8%; *p* < 0.001). ARI prevalence declined progressively with increasing age, from 8.7% among children aged < 12 months to 5.2% among those aged 48–59 months (*p* < 0.001). Male children had a higher prevalence of ARI than female children (7.7% vs 6.7%; *p* < 0.001). Children residing in rural areas experienced a higher burden of ARI than their urban counterparts (7.7% vs 5.8%; *p* < 0.001). Socioeconomic and environmental gradients were also evident. ARI prevalence was highest among children from households in the poorest wealth quintile (8.1%) and decreased steadily with increasing household wealth to 5.4% in the richest quintile (*p* < 0.001). Similarly, children living in households using unclean cooking fuel had a higher prevalence of ARI than those using clean cooking fuel (7.7% vs 6.6%; *p* < 0.001). ARI prevalence was also higher among children of mothers who used tobacco (9.5% vs 7.2%; *p* = 0.002). Children whose mothers had higher education had the lowest prevalence of ARI (5.6%), whereas those whose mothers had no formal education had a prevalence of 7.0% (*p* < 0.001). Although differences in ARI prevalence according to stunting, wasting, and underweight status were relatively small, they were statistically significant (all *p* < 0.001). In contrast, ARI prevalence did not differ significantly by stool disposal practice (7.1% vs 7.3%; *p* = 0.725).

**Table 2:** Prevalence of acute respiratory infection among under-five children in ARI hotspot regions of India, 2019–21

Characteristics	Total N (weighted)	ARI prevalence *n* (%)	95% CI	*p* value
Age of child (months)				
< 12	21,810	1907 (8.74)	8.37–9.12	
12–23	21,116	1846 (8.74)	8.37–9.13	
24–35	21,576	1531 (7.10)	6.76–7.45	< 0.001
36–47	21,378	1386 (6.48)	6.16–6.82	
48–59	22,591	1175 (5.20)	4.92–5.50	
Sex of child				
Male	56,514	4363 (7.72)	7.50–7.94	
Female	51,956	3483 (6.70)	6.49–6.92	< 0.001
Recent diarrhoea				
No	98,991	5709 (5.77)	5.62–5.91	
Yes	9,479	2136 (22.53)	21.70–23.39	< 0.001
Stool disposal practice				
Safe	18,743	1336 (7.13)	6.77–7.51	
Unsafe	89,727	6509 (7.25)	7.09–7.43	0.725
Stunting				
Not stunted	59,891	4371 (7.30)	7.09–7.51	
Stunted	38,219	2889 (7.56)	7.30–7.83	< 0.001
Wasting				
Not wasted	76,313	5659 (7.42)	7.23–7.60	
Wasted	19,879	1526 (7.68)	7.31–8.05	< 0.001
Underweight				
Not underweight	64,764	4808 (7.42)	7.23–7.63	
Underweight	35,454	2643 (7.45)	7.18–7.73	< 0.001
Maternal tobacco use				
No	105,630	7576 (7.17)	7.02–7.33	
Yes	2,840	270 (9.50)	8.47–10.63	0.002
Maternal education				
No education	30,110	2106 (6.99)	6.71–7.29	
Primary	13,178	986 (7.48)	7.05–7.94	
Secondary	49,711	3888 (7.82)	7.59–8.06	< 0.001
Higher	15,472	865 (5.59)	5.24–5.96	
Household wealth quintile				
1st quintile	33,206	2681 (8.07)	7.78–8.37	
2nd quintile	25,445	2015 (7.92)	7.59–8.26	
3rd quintile	19,349	1352 (6.99)	6.64–7.36	< 0.001
4th quintile	16,982	1076 (6.34)	5.98–6.72	
5th quintile	13,489	721 (5.35)	4.98–5.74	
Type of cooking fuel				
Clean	47,850	3154 (6.59)	6.37–6.82	
Unclean	60,621	4691 (7.74)	7.53–7.95	< 0.001
Place of residence				
Urban	24,588	1414 (5.75)	5.47–6.05	
Rural	83,882	6431 (7.67)	7.49–7.85	< 0.001
Overall	108,470	7845 (7.23)	7.08–7.38	

Percentages are weighted. Confidence intervals were estimated using survey-adjusted proportion estimation. *P* values are based on survey-adjusted chi-square tests (Rao–Scott), accounting for sampling weights, clustering, stratification, and strata with single sampling units using centred variance adjustment

### Factors of under-five children’s ARI

**Fig. 3:**
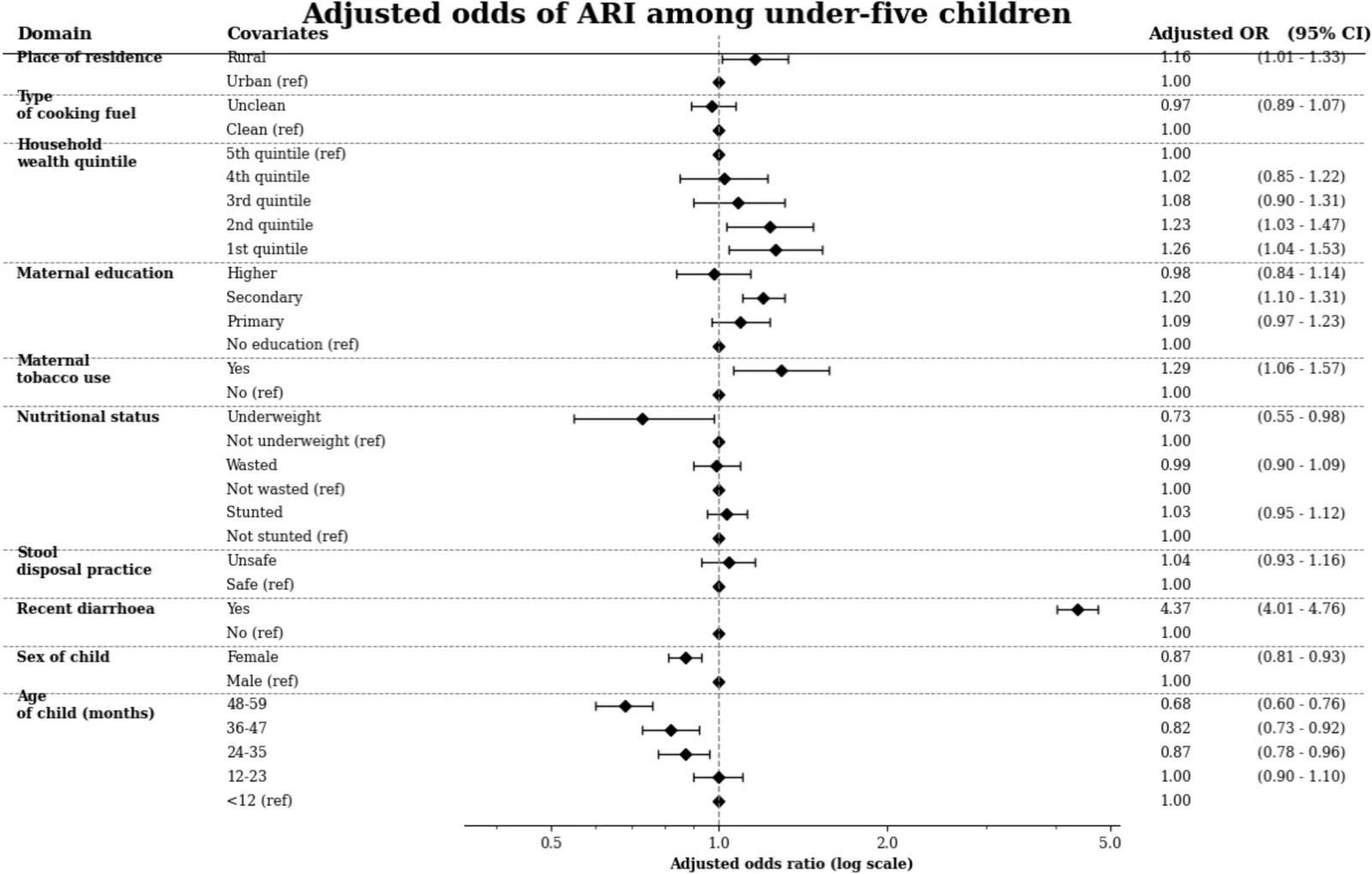
Adjusted odds of acute respiratory infection among under-five children in hotspot regions, India, 2019–21

In the fully adjusted survey-weighted logistic regression model (Model III; weighted *n* = 96,192), several child-, maternal-, and household-level characteristics were associated with ARI (Fig. 3). Compared with children aged < 12 months, those aged 24–35 months (AOR 0.87, 95% CI 0.78–0.96), 36–47 months (AOR 0.82, 95% CI 0.73–0.92), and 48–59 months (AOR 0.68, 95% CI 0.60–0.76) had lower odds of ARI (Table 3). Female children had 13% lower odds than male children (AOR 0.87, 95% CI 0.81–0.93). Recent diarrhoea showed the strongest association (AOR 4.37, 95% CI 4.01–4.76), and maternal tobacco use was also associated with increased odds (AOR 1.29, 95% CI 1.06–1.57). Children in the poorest and second wealth quintiles had higher odds than those in the richest quintile. Rural residence was associated with higher odds (AOR 1.16, 95% CI 1.01–1.33). Stunting, wasting, underweight, stool disposal, and cooking fuel were not independently associated after full adjustment.

**Table 3:** Factors associated with ARI among under-five children in ARI hotspot regions of India, 2019–21

Covariates	Model I AOR (95% CI)	Model II AOR (95% CI)	Model III AOR (95% CI)
Age of child (months)			
< 12 (ref)	1.00	1.00	1.00
12–23	0.99 (0.90–1.10)	0.99 (0.90–1.10)	1.00 (0.90–1.10)
24–35	0.85 (0.77–0.94)***	0.85 (0.77–0.94)**	0.87 (0.78–0.96)**
36–47	0.80 (0.72–0.90)***	0.80 (0.72–0.90)***	0.82 (0.73–0.92)***
48–59	0.66 (0.59–0.74)***	0.66 (0.59–0.74)***	0.68 (0.60–0.76)***
Sex of child			
Male (ref)	1.00	1.00	1.00
Female	0.87 (0.82–0.93)*	0.87 (0.81–0.93)*	0.87 (0.81–0.93)***
Recent diarrhoea			
No (ref)	1.00	1.00	1.00
Yes	4.47 (4.10–4.87)*	4.42 (4.05–4.82)*	4.37 (4.01–4.76)*
Stool disposal practice			
Safe (ref)	1.00	1.00	1.00
Unsafe	1.12 (1.00–1.25)	1.10 (0.99–1.23)	1.04 (0.93–1.16)
Stunting			
Not stunted (ref)		1.00	1.00
Stunted		1.05 (0.97–1.14)	1.03 (0.95–1.12)
Wasting			
Not wasted (ref)		1.00	1.00
Wasted		0.99 (0.90–1.09)	0.99 (0.90–1.09)
Underweight			
Not underweight (ref)		1.00	1.00
Underweight		0.72 (0.54–0.96)	0.73 (0.55–0.98)
Maternal tobacco use			
No (ref)		1.00	1.00
Yes		1.31 (1.08–1.60)*	1.29 (1.06–1.57)*
Maternal education			
No education (ref)		1.00	1.00
Primary		1.07 (0.95–1.20)	1.09 (0.97–1.23)
Secondary		1.12 (1.02–1.22)*	1.20 (1.10–1.31)*
Higher		0.84 (0.72–0.97)*	0.98 (0.84–1.14)
Household wealth quintile			
1st quintile			1.26 (1.04–1.53)*
2nd quintile			1.23 (1.03–1.47)*
3rd quintile			1.08 (0.90–1.31)
4th quintile			1.02 (0.85–1.22)
5th quintile (ref)			1.00
Type of cooking fuel			
Clean (ref)			1.00
Unclean			0.97 (0.89–1.07)
Place of residence			
Urban (ref)			1.00
Rural			1.16 (1.01–1.33)*
Weighted analytic *n*	108,470	96,192	96,192

Estimates were obtained using survey-weighted logistic regression accounting for sampling weights, clustering, stratification, and single-unit strata. Model I used a weighted analytic n of 108,470; Models II and III used a weighted analytic n of 96,192 after complete-case exclusion for missing anthropometric measurements. Reference categories are indicated by “ref”. Multicollinearity was assessed using VIFs

*AOR* adjusted odds ratio, *CI* confidence interval

### Population attributable fractions of ARI

Counterfactual PAF analysis identified recent diarrhoeal illness as the largest potentially preventable contributor among the directly modifiable exposures examined (Table 4). Setting recent diarrhoea to absent for all children yielded a PAF of 19.6% (95% CI 18.1–21.1; *p* < 0.001), whereas setting maternal tobacco use to absent yielded a PAF of 0.7% (95% CI 0.1–1.3; *p* = 0.022). A separate scenario setting rural residence to urban yielded a model-based PAF of 10.2% (95% CI 0.6–18.9; *p* = 0.038). Because residence itself is not directly modifiable and the CI was wide, this estimate is interpreted as a contextual priority-setting indicator representing the bundle of rural disadvantages captured by the model. Simultaneously setting all three exposures to their reference levels yielded a joint PAF of 28.7% (95% CI 20.4–36.1; *p* < 0.001); this was estimated from one joint counterfactual prediction and not by summing individual PAFs.

**Table 4:** Population attributable fractions for ARI among under-five children in ARI hotspot regions of India, 2019–21

Exposure (counterfactual scenario)	PAF (%)	95% CI	*p* value
Recent diarrhoea (no child experienced diarrhoea)	19 · 6	18 · 1–21 · 1	< 0 · 001
Rural residence (all children lived in urban areas)	10 · 2	0 · 6–18 · 9	0 · 038
Maternal tobacco use (no maternal tobacco use)	0 · 7	0 · 1–1 · 3	0 · 022
Combined scenario†	28 · 7	20 · 4–36 · 1	< 0 · 001

Individual and joint PAFs were estimated through counterfactual marginal standardisation after Model III. The joint scenario simultaneously set recent diarrhoea and maternal tobacco use to absent and residence to urban; it was not calculated by adding individual PAFs. Rural residence is a contextual indicator, not a directly modifiable exposure, and the corresponding scenario should be interpreted cautiously

*PAF* population attributable fraction

## Discussion

Using a multistage spatial epidemiological framework and NFHS-5 data, this study identified geographic concentrations, associated factors, and counterfactual burden of ARI among under-five children in India. The unrestricted national prevalence was 2.74%, closely matching the 2.8% reported in another NFHS-5 analysis [8], whereas prevalence was 7.23% after deliberate restriction to statistically identified hotspot districts. This contrast confirms that the hotspot estimate is a high-risk subpopulation estimate rather than a discrepant national estimate. Within hotspots, younger age, recent diarrhoea, maternal tobacco use, poverty, and rural residence were associated with ARI.

Recent diarrhoeal illness was the strongest ARI correlate and had the largest PAF among the directly modifiable exposures. This finding is consistent with prior evidence describing links between recent diarrhoea and respiratory infection among children in Ethiopia, Bangladesh, and other low-resource settings [17–19]. Diarrhoeal episodes and respiratory infections may share determinants, including inadequate water, sanitation, and hygiene, poverty, impaired nutritional status, and reduced immune resilience [20, 21]. The cross-sectional timing of the NFHS measures prevents confirmation that diarrhoea preceded ARI; therefore, the association and PAF should not be interpreted as proof that preventing every diarrhoeal episode would causally avert the estimated proportion of ARI. Nevertheless, the result supports integrated child-health strategies that jointly strengthen diarrhoea prevention, nutrition, and timely management of respiratory symptoms.

Maternal tobacco use remained associated with higher odds of ARI after adjustment for socioeconomic, nutritional, and household characteristics [7]. Although maternal tobacco use was uncommon, its PAF indicated a small but potentially preventable burden in hotspot regions. Tobacco exposure can degrade indoor air quality and is amenable to cessation and household smoke-free interventions. Socioeconomic disadvantage was also important: children from poorer households and rural areas had higher adjusted odds of ARI, plausibly reflecting combined differences in housing, environmental exposures, healthcare access, and care-seeking [2, 17, 20, 21]. Cooking-fuel type and measured nutritional status were not independently associated after adjustment, which may reflect confounding, mediation, exposure misclassification, or limited variation within the hotspot-restricted sample.

The central and eastern concentration observed in NFHS-5 differs from the predominantly northern pattern reported by Balasubramani et al. using NFHS-4 [6]. This apparent spatial shift may reflect genuine changes between survey rounds, including changes in local transmission, household conditions, healthcare access, or reporting. It may also arise from analytical differences, such as district boundaries, inclusion criteria, prevalence estimation, spatial-weighted specifications, and significance thresholds. Because the two analyses were not harmonised within a single spatiotemporal model, the present findings should not be interpreted as definitive evidence of geographic migration of ARI risk. A harmonised district-level comparison across NFHS rounds is needed to distinguish epidemiological change from methodological variation.

## Strengths and limitations

This study has several strengths. It uses a large national survey and a two-stage framework combining district-level hotspot identification, survey-weighted regression, and counterfactual PAF estimation. Reporting both the unrestricted national prevalence and the hotspot-restricted prevalence clarifies the estimands. Restricting the second-stage analysis to empirically identified hotspots provides evidence relevant to geographic prioritisation, while the survey design was incorporated in estimation and inference.

Several limitations should be acknowledged. The cross-sectional design precludes establishing temporality or causality. ARI and diarrhoea were based on maternal recall during the preceding 2 weeks and may be misclassified. Clinical severity and aetiology were unavailable. Ambient air pollution, ventilation, seasonality, immunisation, and other contextual factors may leave residual confounding. Complete-case regression excluded children with missing anthropometric measurements; if missingness was not random, estimates may be biased. Hotspot selection based on the outcome produces a deliberately high-prevalence subpopulation and limits generalisation of second-stage estimates to hotspot districts. PAF estimates depend on model specification, consistency, positivity, and absence of uncontrolled confounding and, therefore, describe model-based potential reductions, not guaranteed intervention effects. In particular, rural residence is not directly modifiable and its wide PAF interval warrants caution.

## Policy implications

The findings of this study have direct implications for child-health policy and programme implementation in India. The identification of distinct ARI hotspot regions indicates that uniform, nationwide approaches are insufficient to reduce the burden of under-five ARI. Instead, geographically targeted strategies that prioritise high-burden districts are essential to maximise health gains and optimise resource allocation.

First, the strong association between recent diarrhoeal illness and ARI, together with the large population attributable fraction, underscores the need for integrated child-health interventions. Strengthening convergence between diarrhoea control programmes and respiratory infection prevention within the Integrated Management of Neonatal and Childhood Illness framework could substantially reduce ARI burden in hotspot regions.

Second, the independent contribution of maternal tobacco use highlights an overlooked but modifiable risk factor for childhood ARI. Tobacco control initiatives, including the National Tobacco Control Programme, should explicitly integrate maternal and household-level cessation counselling into routine maternal and child-health services, particularly in ARI hotspot districts. Community-based platforms such as Integrated Child Development Services and village health and nutrition days offer feasible entry points for such interventions.

Third, the association with rural residence should guide place-based investment rather than be interpreted as a recommendation to change residence. Relevant policy levers include strengthening rural primary healthcare, timely referral, clean household energy, ventilation, water and sanitation, immunisation, and access to early treatment. Hotspot districts may be prioritised for enhanced outreach through Ayushman Bharat Health and Wellness Centres.

Overall, embedding spatial epidemiological evidence into national and state-level planning can improve targeting, efficiency, and equity of interventions, supporting India’s progress toward Sustainable Development Goal 3.2-ending preventable deaths of children under 5 years by 2030.

## Conclusion

Under-five ARI in India was concentrated in geographically distinct hotspot districts, predominantly across central and eastern regions. The hotspot prevalence of 7.23% was substantially higher than, but conceptually distinct from, the unrestricted national prevalence of 2.74%. Recent diarrhoeal illness accounted for the largest model-based preventable fraction, while maternal tobacco use contributed a smaller fraction. Rural residence identified place-based disadvantage but is not itself a directly modifiable exposure; its PAF should, therefore, be used cautiously for priority setting. Integrated diarrhoea prevention, tobacco cessation, and investments in rural child-health infrastructure should be prioritised in hotspot districts. These cross-sectional PAF estimates are hypothetical and should inform, rather than be equated with, expected intervention effects.

## Data Availability

No datasets were generated or analysed during the current study.
